# Reduction of DILP2 in *Drosophila* Triages a Metabolic Phenotype from Lifespan Revealing Redundancy and Compensation among DILPs

**DOI:** 10.1371/journal.pone.0003721

**Published:** 2008-11-13

**Authors:** Susan Broughton, Nazif Alic, Cathy Slack, Timothy Bass, Tomoatsu Ikeya, Giovanna Vinti, Anna Maria Tommasi, Yasmine Driege, Ernst Hafen, Linda Partridge

**Affiliations:** 1 UCL Institute of Healthy Ageing, GEE, University College London, London, United Kingdom; 2 ETH Zurich, Institute of Molecular Systems Biology, Zurich, Switzerland; Baylor College of Medicine, United States of America

## Abstract

The insulin/IGF-like signalling (IIS) pathway has diverse functions in all multicellular organisms, including determination of lifespan. The seven insulin-like peptides (DILPs) in *Drosophila* are expressed in a stage- and tissue-specific manner. Partial ablation of the median neurosecretory cells (mNSCs) in the brain, which produce three DILPs, extends lifespan, reduces fecundity, alters lipid and carbohydrate metabolism and increases oxidative stress resistance. To determine if reduced expression of DILPs is causal in these effects, and to investigate possible functional diversification and redundancy between DILPs, we used RNA interference to lower specifically the transcript and protein levels of *dilp2*, the most highly expressed of the mNSC-derived DILPs. We found that DILP2 was limiting only for the increased whole-body trehalose content associated with mNSC-ablation. We observed a compensatory increase in *dilp3* and *5* mRNA upon *dilp2* knock down. By manipulation of *dfoxo* and *dInR*, we showed that the increase in *dilp3* is regulated via autocrine insulin signaling in the mNSCs. Our study demonstrates that, despite the correlation between reduced *dilp2* mRNA levels and lifespan-extension often observed, DILP2 reduction is not sufficient to extend lifespan. Nor is the increased trehalose storage associated with reduced IIS sufficient to extend lifespan. To understand the normal regulation of expression of the *dilp*s and any functional diversification between them will require independent control of the expression of different *dilp*s.

## Introduction

The insulin/IGF-like signalling (IIS) pathway, present throughout multicellular animals [1], has functions including the regulation of growth, development [2]–[5] and metabolic homeostasis [6], as well as determination of adult lifespan, resistance to stress and fecundity, in *C. elegans*, *Drosophila* and mouse [8]–[15]. Mutations that reduce the activity of IIS can increase lifespan in all three organisms, often in conjunction with associated alterations in growth, stress resistance, metabolic phenotypes and fecundity. Identifying the precise modulations of IIS that are required for lifespan-extension is thus important for determining which, if any, of the associated phenotypes are either causal in extension of lifespan or unavoidably associated with it.

Intracellular components of IIS are encoded by single genes in the invertebrates *C. elegans* and *Drosophila*, with the exception of the triplication of the protein kinase B, *Akt*, SGK-1 in *C. elegans* [16], resulting in a relatively simple intracellular signalling pathway. In contrast, mammals have several versions of the intracellular components of IIS. On the other hand, there are multiple genes for the insulin-like ligands in the two invertebrates, with seven detected in the *Drosophila* genome [3] and 38 in *C. elegans* [16]. The seven *Drosophila* insulin-like peptides (DILPs) are predicted to resemble preproinsulin at the structural level, and are therefore considered orthologous to mammalian insulin. The genes encoding the *Drosophila* insulin-like peptides are independently transcriptionally regulated in response to nutrition, as well as in a tissue- and stage-specific manner during development [3], [17]. Thus, in these invertebrates, the multiple functions of IIS may be mediated in part by functional diversification of the ligands.

Reducing the levels of a subset of the DILPs, by ablation of DILP2, 3 and 5-producing mNSCs in the *pars intercerebralis* of the brain late in the final larval instar, leads to an array of phenotypes including increased fasting glucose levels in the adult hemolymph, increased storage of lipid and carbohydrate, reduced fecundity, extension of median and maximal lifespan and increased resistance to oxidative stress and starvation [14]. Ablation of these cells earlier in larval development resulted in developmental delay, growth retardation, and elevated carbohydrate levels in larval hemolymph [4]. These findings imply that reduction in the level of one or more of the three DILPs produced in these cells causes this diverse array of phenotypes, but direct proof of this, together with information on possible functional diversification and redundancy of the DILPs, requires direct manipulation of the levels of individual DILPs.

Of the DILPs produced by the mNSCs, DILP2 is currently thought to be the most important. It is the most highly expressed, the most potent growth stimulator and over-expression of it alone can rescue the diabetic phenotypes of mNSC-ablated larvae [14], [4], [17]. Furthermore, DILP2 has been suggested to play a prominent role in lifespan-extension by reduced IIS [18]–[21]. Hwangbo et al [18], and more recently Min et al [21], reported a reduction in *dilp2*, but not in *dilp3* and *5* mRNA, in response to activated FOXO in head fat body, and proposed that this reduction mediated lifespan-extension. *dilp2* expression also responds to JNK activation in the mNSCs, and the resulting lifespan-extension was suggested to be mediated by Foxo-dependent repression of *dilp2* [19]. Bauer et al [20] showed that expression of a dominant negative form of p53 in adult neurons extended lifespan and reduced *dilp2* transcript levels, and again suggested that the reduction of *dilp2* expression was responsible for the increase in lifespan. However, none of these studies experimentally manipulated *dilp2* expression and nor did they establish whether DILP protein levels were affected by the changes in transcript level.

To test experimentally the hypothesis that a reduction in *dilp2* alone produces lifespan extension, and to ascertain if other phenotypes regulated by DILP2 could be determinants of lifespan, we reduced *dilp2* expression using RNAi specifically against *dilp2* in the mNSCs, and examined the consequences for lifespan-extension and other phenotypes associated with mNSC ablation. We found that reducing *dilp2* alone in the adult fly to a level similar to that due to mNSC ablation and observed in the above-mentioned studies correlating *dilp2* levels and lifespan [18]–[21], had no effect on lifespan, fecundity, stress resistance, hemolymph carbohydrate levels or glycogen levels. A possible explanation of this finding is compensation by increased expression of one or more of the other *dilps*, if there can be functional redundancy between them. Indeed, increases in *dilp3* and *5* mRNA were observed following *dilp2* knock down, suggesting compensatory regulation of *dilp3* and *5*. In the case of *dilp3*, this up-regulation may be due to reduced IIS in the mNSCs upon *dilp2* knock down since we could show that *dilp3* is up-regulated upon down regulation of IIS via manipulation of the insulin receptor activity and that it requires FOXO for its basal expression. Knock down of *dilp2* did, however, lead to an increase in total trehalose content of the same magnitude as that resulting from mNSC ablation. *dilp2* expression is therefore not limiting for lifespan, and it plays an individual role only in the increase in trehalose storage among the phenotypes affected by mNSC ablation. Furthermore, our data show that increased trehalose storage alone is not sufficient to extend lifespan.

## Results

### Targeted knock-down of *dilp2* by RNAi

**Figure 1:**
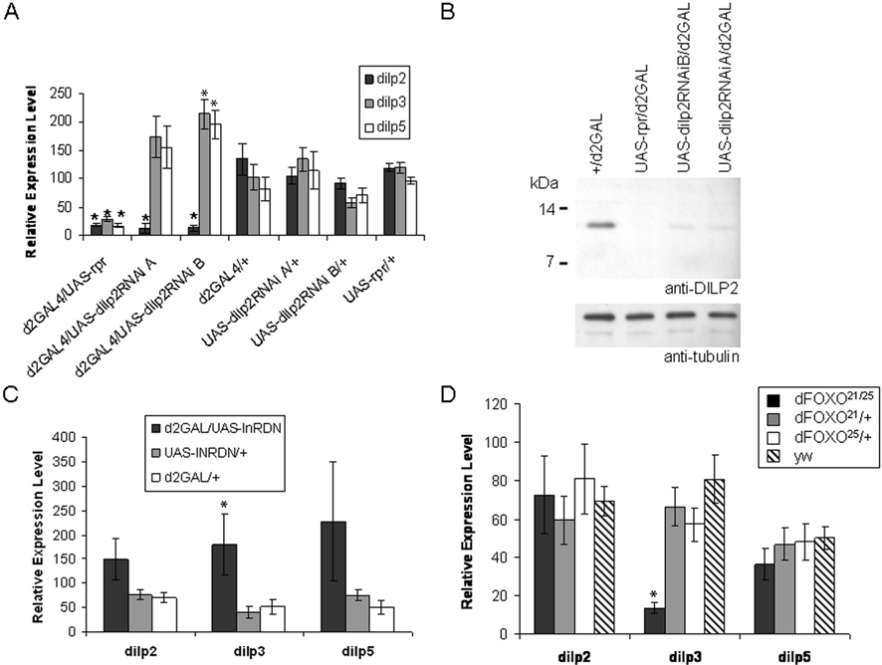
Characterization of DILP2 knock-down in UAS-dilp2RNAi/d2GAL flies. The effect of UAS-dilp2RNAi driven by d2GAL in the mNSCs on *dilp* expression in 7 day old adult female heads was measured by quantitative RT-PCR and Western blot analysis. (A) *dilp 2, 3* and *5* relative transcript levels in dilp2RNAi/d2GAL and control female heads. (B) Western blot analysis of DILP2 (top panel) or the tubulin loading control (bottom panel) in total head protein extracted from females of the indicated genotype. (C) *dilp 2, 3* and *5* relative transcript levels in d2GAL/UAS-InR^DN^ and control female heads. (D) *dilp 2, 3* and *5* relative transcript levels in a FOXO null mutant (FOXO^21/25^) and control female heads. (A, C and D): data are shown as mean relative expression±SEM (N = 5), * denotes significant difference to controls (P<0.05).

To examine the role of DILP2 in lifespan extension and other phenotypes associated with mNSC ablation, we directed RNAi specifically against *dilp2* in the mNSCs–the only cells expressing *dilp2* in the adult [14; FlyAtlas], [22]. We generated two independent insertion lines (A and B) of a UAS-dilp2RNAi transgene and drove its expression in the mNSCs using d2GAL, a GAL4 transgene with expression directed by a fragment of the *dilp2* promoter [17], which we have previously used to ablate the mNSCs in the adult [14]. Relative *dilp* transcript levels in adult female heads were reduced by approximately 80% of control levels in the UAS-dilp2RNAi/d2GAL genotypes (Figure 1A), a reduction similar to that observed in mNSC-ablated flies and greater than that due to fat body over-expression of dFOXO, JNK activation in mNSCs, or over-expression of dominant negative p53 in mNSCs [18]–[21].

We confirmed that RNAi also reduced DILP2 protein levels. We separated the protein extracts from heads on non-reducing Tris-Tricine gels, and performed a western blot (Figure 1B) using an anti-DILP2 antibody that specifically recognises the mNSCs [14]. A protein of apparent molecular weight of 12 kDa was observed in the controls but was not detectable in the mNSC-ablated (d2GAL/UAS-rpr) flies. This protein could not have been DILP3 or 5 since the sequence of the peptide used to generate the anti-DILP2 antibody (CEEYNPVIPH) is unique to DILP2. DILP2 was detectable in the dilp2RNAi/d2GAL genotypes but was reduced to approximately 10% of control levels, confirming DILP2 knock-down. Note that the band observed corresponds in size to pro-DILP2, and that we could not detect any processed DILP2.

### Knock-down of *dilp2* results in up-regulation of *dilp3* and *5*, which for *dilp3* may occur through reduction of insulin signalling in the mNSCs

RNAi for *dilp2* in the mNSCs could alter levels of *dilp3* and *dilp5*, either by knocking them down (as has been observed for an independent *dilp3* RNAi construct [21]) due to sequence homology, or by a compensatory up-regulation of expression of *dilp3* and *5*. We confirmed that the knock-down was specific to *dilp2*, because no decrease in *dilp3* and *5* transcripts was observed in either line. On the contrary, we observed increased levels of *dilp3* and *5* transcripts, which were statistically significant in line B (Figure 1A).

To investigate if these apparently compensatory increases in *dilp*3 and 5 mRNA were due to an autocrine feedback loop, we examined the levels of *dilp2, 3* and *5* in flies expressing a dominant-negative form of the *Drosophila* insulin receptor (UAS-InR-DN) in the mNSCs. This dampening of the insulin signal in the mNSCs resulted in a significant increase in *dilp3* mRNA levels, paralleled with more variable increases in *dilp2* and *5* mRNA (Figure 1C). That *dilp3* transcription is regulated by insulin signalling was further confirmed by the observation that FOXO is required for basal levels of *dilp3* expression, since *dilp3* transcript was significantly reduced in FOXO null flies (Figure 1D). Deletion of FOXO did not, however, affect the levels of *dilp2* or *5* mRNA. Indeed, by searching for perfect matches to the mouse Foxo1/Foxo4 consensus binding site (RWWAACA) 1 Kb upstream of the ATG we could identify eight putative FOXO binding sites in the *dilp3* promoter compared with two and one for the *dilp2* an*d 5* promoters, respectively. Hence, reduction in *dilp2* by RNAi in the mNSCs causes up-regulation of *dilp3* and *5* transcription that, in the case of *dilp3*, appears to occur via direct autocrine regulation through the insulin signalling pathway.

### dilp2RNAi/d2GAL flies are not longer-lived or less fecund than controls

**Figure 2:**
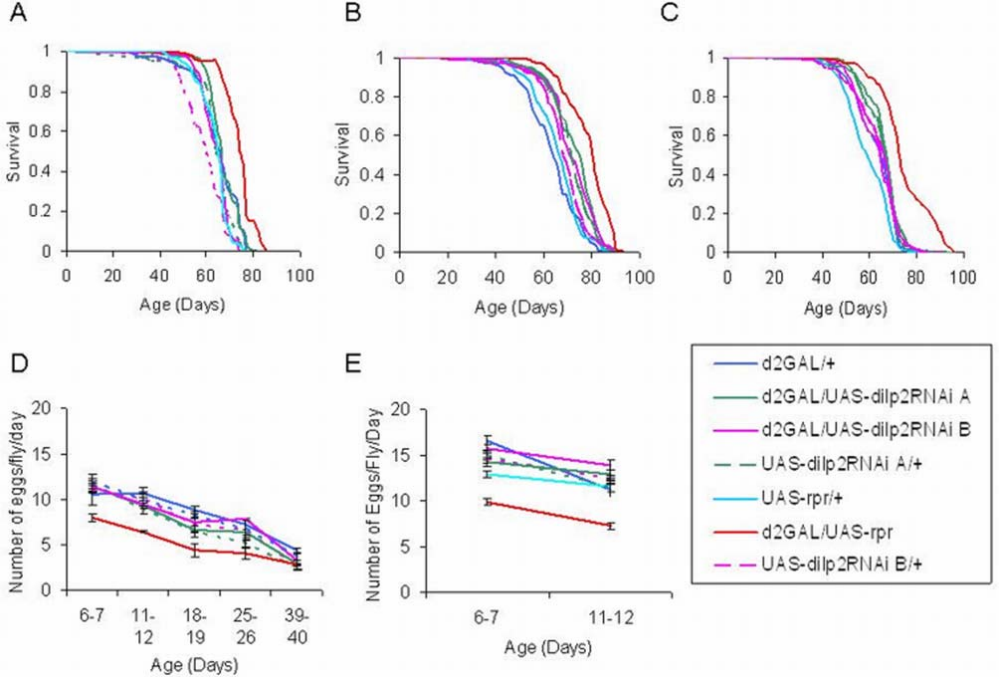
dilp2RNAi/d2GAL mated females are not long-lived or less fecund than controls. Survival curves, median lifespans, percentage increase compared to control, and sample sizes are as follows. (A): Experiment 1. Median lifespan of: UAS-rpr/d2GAL = 75 days (13.6% increase over UAS-*rpr*/+ & d2GAL/+ controls, P<0.0001), N = 69; UAS-dilp2RNAiA/d2GAL = 67 days, N = 71; UAS-dilp2RNAiA/+ = 66 days, N = 36; UAS-dilp2RNAiB/d2GAL = 66 days, N = 130; UAS-dilp2RNAiB/+ = 60 days, N = 92; UAS-rpr/+ = 66 days, N = 110; and d2GAL/+ = 66 days, N = 61. (B) Experiment 2. Median lifespan of: UAS-rpr/d2GAL = 81 days (19% increase over UAS-*rpr*/+ control, P<0.0001), N = 155; UAS-dilp2RNAiA/d2GAL = 76 days, N = 157; UAS-dilp2RNAiA/+ = 73 days, N = 137; UAS-dilp2RNAiB/d2GAL = 71 days, N = 145; UAS-dilp2RNAiB/+ = 69 days, N = 129; UAS-rpr/+ = 68 days, N = 139; and d2GAL/+ = 64 days, N = 153. (C) Experiment 3. Median lifespan of: UAS-rpr/d2GAL = 73 days (12% increase over d2GAL/+ control, P<0.0001), N = 153; UAS-dilp2RNAiA/d2GAL = 68 days, N = 180; UAS-dilp2RNAiA/+ = 67 days, N = 166; UAS-dilp2RNAiB/d2GAL = 65 days, N = 179; UAS-dilp2RNAiB/+ = 67 days, N = 175; UAS-rpr/+ = 59 days, N = 149; and d2GAL/+ = 65 days, N = 169. (D) Fecundity of females from experiment 1 shown in (A). (E) Fecundity of females from experiment 2 shown in (B). Data are shown as mean number of eggs laid per female per day±SEM.

In two independent experiments, reduced *dilp2* had no significant effect on lifespan (Figure 2A, B) or fecundity (Figure 2D, E) under standard conditions. As the response of lifespan and *dilp2* levels to fat body expression of FOXO depended on nutrient conditions [21], we assessed the effect of increased yeast concentration on lifespan in the dilp2RNAi and mNSC-ablated flies. Lifespan was measured on food with 1.5x normal yeast concentration (150 g/l), and again no effect of reduction of *dilp2* alone on lifespan was found, although the mNSC-ablated flies were still longer lived than their controls (Figure 2C). The magnitude of lifespan extensions due to mNSC-ablation in the three experiments is within the range of those seen previously (14 and data not shown). Hence, although DILP2 was reduced to very low levels, this reduction was not sufficient to produce the lifespan and fecundity phenotypes of the mNSC-ablated flies, suggesting that DILP2 levels are not limiting for these phenotypes. Furthermore, in contrast to mNSC ablation, the reduction in DILP2 alone had no effect on growth as indicated by adult weight (data not shown).

### dilp2RNAi/d2GAL flies are not resistant to oxidative stress

**Figure 3:**
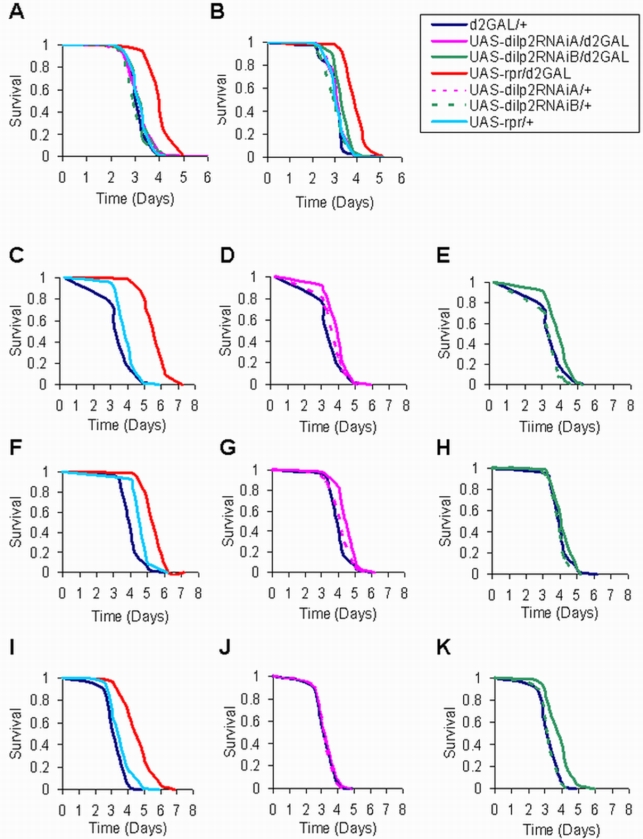
Survival of dilp2RNAi/d2GAL female flies under oxidative stress and starvation. Survival curves, median lifespans, percentage increase compared to control, and sample sizes of 7 day old mated females are as follows: (A) Experiment 1 on 5% H_2_O_2_. Median lifespans (in days) of: UAS-rpr/d2GAL = 4.04, N = 104, (34% increase over d2GAL/+ control, P<0.0001); UAS-dilp2RNAiA/d2GAL = 3.25, N = 102; UAS-dilp2RNAiA/+ = 3.25, N = 100; UAS-dilp2RNAiB/d2GAL = 3.25, N = 77; UAS-dilp2RNAiB/+ = 2.9, N = 79; UAS-rpr/+ = 3.01, N = 82; and d2GAL/+ = 3.01, N = 98. (B) Experiment 2 on 5% H_2_O_2_. Median lifespans (in days) of: UAS-rpr/d2GAL = 3.85, N = 77, (15.6% increase over d2GAL/+ control, P<0.0001); UAS-dilp2RNAiA/d2GAL = 3.21, N = 83; UAS-dilp2RNAiA/+ = 3, N = 96; UAS-dilp2RNAiB/d2GAL = 3.33, N = 58; UAS-dilp2RNAiB/+ = 3.21, N = 84; UAS-rpr/+ = 3.21, N = 91; and d2GAL/+ =  3.33, N = 87. (C–E) Experiment 1 on 1% agar. Median lifespans (in days) of: UAS-rpr/d2GAL (mNSC-ablated) = 5.9, N = 120, (47.5% increase over UAS-rpr/+ & d2GAL/+ controls, P<0.0001); UAS-dilp2RNAiA/d2GAL = 4.17, N = 112, (4.25% increase over UAS-Dilp2RNAiA/+ control, P<0.0001); UAS-dilp2RNAiA/+ = 4, N = 115; UAS-dilp2RNAiB/d2GAL = 4.17, N = 98, (4.25% increase over d2GAL/+ control, P = 0.0004); UAS-dilp2RNAiB/+ = 3.86, N = 117; UAS-rpr/+ = 4, N = 116; and d2GAL/+ = 4, N = 117. (F–H) Experiment 2 on 1% agar, Median lifespans (in days) of: UAS-rpr/d2GAL = 6.2, N = 91, (25% increase over UAS-rpr/+ control, P<0.0001); UAS-Dilp2RNAiA/d2GAL = 4.96, N = 94, (19.5% increase over UAS-dilp2RNAiA/+ control, P = 0.0041); UAS-dilp2RNAiA/+ = 4.15, N = 91; UAS-dilp2RNAiB/d2GAL = 4.15, N = 88; UAS-dilp2RNAiB/+ = 3.96, N = 91; UAS-rpr/+ = 4.96, N = 98; and d2GAL/+ = 3.96, N = 98. (I–K) Experiment 3 on 1% agar, Median lifespans (in days) of: UAS-rpr/d2GAL = 4.85, N = 89, (24% increase over UAS-rpr/+ control, P<0.0001); UAS-dilp2RNAiA/d2GAL = 3.25, N = 85; UAS-dilp2RNAiA/+ = 3.17, N = 94; UAS-dilp2RNAiB/d2GAL = 3.9, N = 76, (23% increase over d2GAL/+ control, P<0.0001); UAS-dilp2RNAiB/+ = 3.06, N = 76; UAS-rpr/+ = 3.9, N = 85; and d2GAL/+ = 3.17, N = 82.

mNSC ablation results in oxidative stress resistance [14], and DILP2 has been postulated to play a role in the response to oxidants [19]. We therefore examined tolerance to H_2_O_2_ and found that, while the mNSC-ablated flies were resistant, the dilp2RNAi/d2GAL flies were not (Figure 3A and B). Hence, the reduction in DILP2 alone was not sufficient to increase tolerance to oxidative stress. It should be noted that the mNSC-ablated flies were not resistant to paraquat in the current study (data not shown) although they had been shown previously to be resistant to this oxidant [14]. This difference may have resulted from the change in the food used for fly rearing and experiments, as is discussed further below.

### The stored glycogen, lipid and fasting hemolymph carbohydrate phenotypes of the mNSC-ablated flies are not affected by dilp2RNAi, but dilp2RNAi flies do contain higher levels of stored trehalose

**Figure 4:**
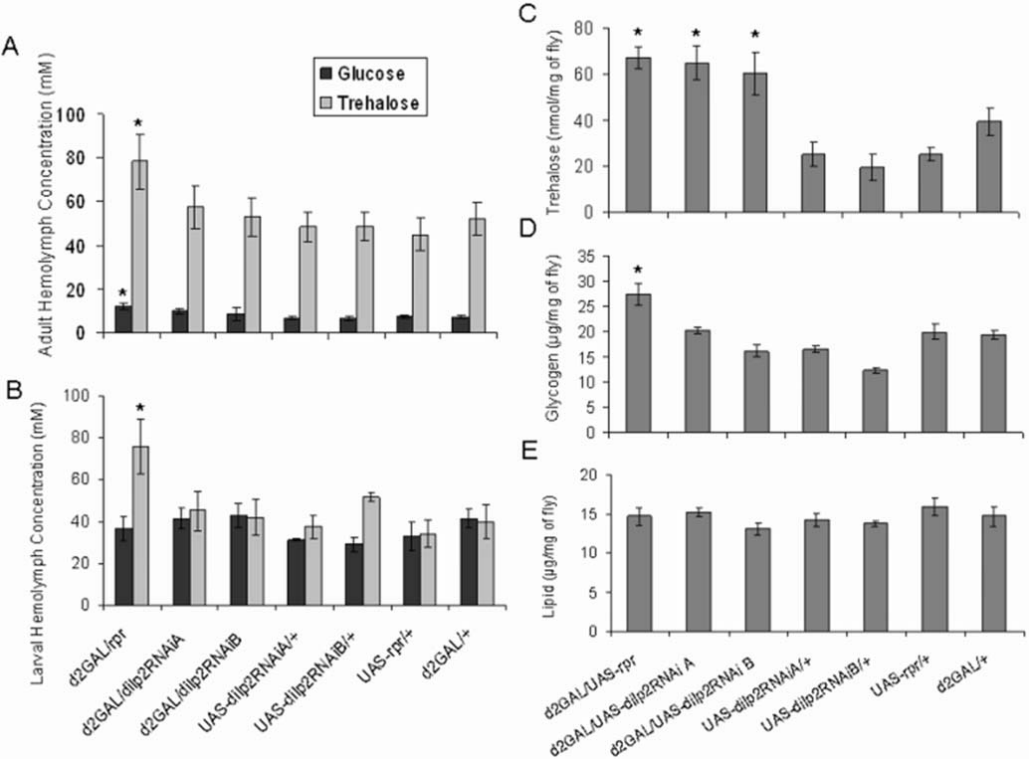
The effect of dilp2RNAi expression in mNSCs on hemolymph glucose and trehalose levels, and whole-body trehalose, glycogen and lipid content. (A) Hemolymph glucose and trehalose concentrations in 7 day old mated females, maintained on standard food with 100 g/l of sugar and fasted on 1% agar for 5 hours prior to testing. N = 10 for each genotype. (B) Hemolymph glucose and trehalose concentrations in third instar wandering larvae that developed on standard food with 100 g/l of sugar: UAS-rpr/d2GAL (N = 7), UAS-dilp2RNAiA/d2GAL (N = 7), UAS-dilp2RNAiA/+ (N = 5), UAS-dilp2RNAiB/d2GAL (N = 6), UAS-dilp2RNAiB/+ (N = 6), UAS-rpr/+ (N = 5) and d2GAL/+ (N = 6). (C) Whole-fly trehalose content per mg of fly (fresh weight). UAS-rpr/d2GAL (N = 20), UAS-dilp2RNAiA/d2GAL (N = 20), UAS-dilp2RNAiA/+ (N = 10), UAS-dilp2RNAiB/d2GAL (N = 20), UAS-dilp2RNAiB/+ (N = 10), UAS-rpr/+ (N = 10) and d2GAL/+ (N = 20). (D) Glycogen content per mg of fly (fresh weight), N = 21. (E) Lipid content per mg of fly (fresh weight), N = 17. In all panels, data are shown as mean±SEM, * indicates significant difference to appropriate controls (P<0.05).

We previously showed that mNSC-ablated flies exhibited generally higher levels of stored energy (trehalose, glycogen and lipid) and an altered profile of adult, fasting, circulating carbohydrates compared with controls [14]. Neither fasting hemolymph trehalose and glucose levels in adults and larvae nor glycogen levels in adult whole body extracts were increased in the dilp2RNAi/d2GAL genotypes (Figure 4A, B, and D). Reduction in DILP2 alone was therefore not sufficient to raise hemolymph carbohydrate or stored glycogen levels. The reduction in DILP2 alone was, however, sufficient to raise levels of stored trehalose similarly to that due to mNSC ablation. The dilp2RNAi/d2GAL genotypes were found to contain significantly higher levels of trehalose in whole body extracts relative to body mass than controls (Figure 4C). The total volume of hemolymph in an adult fly is extremely small, approximately 0.1 µl [23], such that the contribution of any hemolymph trehalose to the total trehalose content can be regarded as negligible. Thus, reduction in DILP2 alone was sufficient to increase total fly trehalose by the same magnitude as mNSC ablation, suggesting that the increase in total trehalose observed in the mNSC-ablated flies may be mediated solely by the reduction in DILP2.

Although unlikely, it was possible that the activation of the RNAi machinery itself in the mNSCs due to expression of our dilp2RNAi transgene may have had an effect on trehalose metabolism. We therefore measured the effect of expression of UAS-GFP-RNAi driven by d2GAL in the mNSCs and confirmed that trehalose levels were not affected (Figure S1), further supporting the conclusion that it was the reduction in DILP2 that was causal in the increased trehalose phenotype.

It should be noted that the increase in lipid observed previously due to mNSC-ablation [14] was not observed in the current study in the mNSC-ablated or dilp2RNAi flies (Figure 4E). In addition, the increased hemolymph sugar phenotype of the mNSC-ablated flies was slightly different to that seen previously (Figure 4A). The alteration of these metabolic phenotypes may be due to the yeast used or the sucrose content in the food for the current experiments, both of which have been optimised for lifespan measurements [24]. The yeast used in this study is from a different supplier than that used previously, and the nutritional make-up of yeasts from different suppliers varies greatly resulting in different effects on lifespan and fecundity [24]. The metabolic phenotypes in the ablated flies appear to be sensitive to the nutrient content of the food, and the same may account for the loss of the paraquat resistance phenotype in the mNSC-ablated flies, where differences in the total antioxidant activity of the yeasts may have resulted in a difference in paraquat tolerance.

### dilp2RNAi/d2GAL flies display a very slight starvation resistance

The increase in whole body trehalose content observed on DILP2 reduction and the fact that the mNSC-ablated flies are starvation resistant [14] prompted us to examine the starvation sensitivity of the dilp2RNAi flies. In the current study, the mNSC-ablated flies (UAS-rpr/d2GAL) were again consistently long-lived displaying a 24% to 47% increase in median lifespan over controls in three starvation trials (P<0.0001). The dilp2RNAi/d2GAL genotypes both displayed a small extension of median, but not maximum, lifespan in two out of three starvation trials (Figure 3). The dilp2RNAiA/d2GAL genotype showed a significant increase in median lifespan in trial 1 (Figure 3D, 4.25% greater than d2GAL/+ control, P<0.0001) and in trial 2 (Figure 3G, 19.5% greater, P = 0.0041). In trial 3 there was no significant difference to controls (Figure 3J). The dilp2RNAiB/d2GAL genotype showed a significant increase in median lifespan in trial 1 (Figure 3E, 4.25% greater than d2GAL/+ control, P<0.0004) and in trial 3 (Figure 3K, 23% greater than d2GAL/+ control, P<0.0001). In trial 2 there was no significant difference to controls (Figure 3H). Although the effect was small and variable, it suggests that a decrease in DILP2 may partially mediate starvation resistance due to mNSC ablation.

## Discussion

*dilp2* is the most highly expressed of the mNSC-derived *dilp*s, and several studies have indicated its functional significance and the prominent role it may play in mediating lifespan-extension due to reduced IIS [18]–[21], prompting us to investigate the role of DILP2 directly. We were successful in specifically reducing both the levels of RNA and protein, and found that DILP2 levels were limiting for only one of the phenotypes resulting from mNSC ablation, increased whole body trehalose content. It remains quite possible that the lifespan extensions due to the FOXO, JNK or p53 manipulations [18]–[21] were mediated by reductions in one or more of DILPs 2, 3 and 5, because the context in which the level of *dilp2* was lowered was different in each case. This could, for instance, have resulted in different levels of transcript for other *dilp*s or different states of the intracellular IIS pathway. Our findings do show clearly, however, that neither reduction in *dilp2* RNA and DILP2 protein expression alone nor increased trehalose storage alone is sufficient to extend lifespan.

Of the phenotypes observed on mNSC-ablation, we found that reduction in DILP2 had an effect only on whole body trehalose levels. This result could indicate that reduction in DILP2 *per se* is required for increased stored tehalose upon mNSC-ablation. Alternatively, if there is functional redundancy between the 3 DILPs produced in the mNSCs, it is possible that trehalose storage is the most sensitive of the phenotypes to a reduction in the overall expression of DILPs in the mNSCs. The compensation that we observed in transcript levels of *dilp*s *3* and *5* upon reduction in *dilp2* transcript may not be sufficient to bring overall *dilp* transcript levels in the mNSCs back to normal, because *dilp2* is much more highly expressed than the other two [14], [18]. Similar arguments apply to the lack of effect of reduction in DILP2 levels on the other phenotypes associated with mNSC-ablation. Because of the compensation in *dilp*s 3 and 5, the result could indicate that there is functional redundancy between DILPs and that there was insufficient reduction in overall DILP levels for these phenotypes to appear. Consistent with redundancy, while we have shown that DILP2 levels are not limiting for the hemolymph carbohydrate phenotype (Figure 3), over-expression of *dilp2* alone can rescue the growth and hemolymph carbohydrate phenotypes due to early ablation of the mNSCs [4]. Alternatively DILP2 may not be involved in producing these phenotypes when the mNSCs are ablated, other than the increased whole body trehalose. To determine which interpretation is correct would require independent manipulation to varying degrees of the different *dilp*s.

The increases in *dilp3* and *5* transcripts when *dilp2* is knocked down suggest that there may be compensatory increases in DILP3 and 5 proteins. It is interesting in this respect that our data show that *dilp3* is the only *dilp* in the mNSCs whose expression is sensitive to reduced insulin signaling in these cells, implicating an autocrine feedback loop. The other two *dilp*s may be regulated in response to other signals, such as nutritional status in the case of *dilp5* [21]. Hence, different *dilp*s may be produced in response to different intrinsic/extrinsic stimuli but once produced function redundantly.

A specific role for DILP2 in trehalose metabolism raises the possibility of distinct roles for DILPs 3 and 5 in one or more of the phenotypes of the ablated flies, including lifespan. Although all seven DILPs are capable of promoting growth [3] and thus acting redundantly in this circumstance, the phenotypes resulting from a lowering of a subset of the DILPs [2], [Bibr pone.0003721-Brogiolo1] [3 and 5] despite the persistence of the remaining ligands suggests at least some functional specificity among them. This notion is supported by the finding that the abolition of sexual dimorphism in locomotor behaviour, which is a consequence of mNSC ablation in males, unlike growth or hemolymph sugars, cannot be rescued by injection or over-expression of DILP2 alone [25]. In addition, *Drosophila* p70/S6 kinase in the mNSCs mediates hunger regulation of feeding behavior in larvae, and over-expression of DILPs 2 and 4, but not DILP3, suppresses this hunger-driven behavior [26]. The specificity of the DILPs may be determined by their biochemical properties, regulation of their synthesis as well as their sites of release.

The physiological role of the regulation of trehalose metabolism is not known. Increased whole-body trehalose has been correlated with resistance to anoxia [27]. We, however, could not observe such resistance in the DILP2-knock-down lines or the mNSC-ablated flies (data not shown). The increased trehalose in the dilp2RNAi/d2GAL flies correlated with a slight increase in resistance to starvation, indicating that these trehalose stores play, if any, only a minor part in starvation tolerance. Furthermore, this observation also indicates that the starvation resistance of the mNSC-ablated flies does not stem from the increased whole-body trehalose. These data are consistent with a recent finding that *Drosophila* ARC protein, which is expressed in the *dilp*-producing mNSCs, is a regulator of behavioural responses to starvation but is not a general regulator of insulin signalling [28]. Mutants are starvation resistant likely due to their loss of normal starvation induced hyperlocomotion. It is therefore possible that the mNSC-ablated flies are starvation resistant predominantly because of a reduction in ARC, and the slight effect on starvation resistance following DILP2 knock down in the dilp2RNAi flies may be due to an alteration of metabolic rates and the consumption and distribution of energy sources, of which increased whole body trehalose may be a sign.

Further investigation of putative specific roles of the individual DILPs, which awaits production of specific mutants or effective RNAi against *dilps 3* and *5,* may shed light on the links between the different aspects of fly physiology they control. However, our finding that DILP2 levels are not limiting for lifespan, fecundity and stress resistance clearly demonstrates that we need to change our thinking about how *dilp*s regulate lifespan and other traits, and we need direct experimental manipulation to address this issue.

## Materials and Methods

### Fly Stocks and Maintenance

The control *white* ^Dahomey^ background stock, UAS-*reaper* and *dilp2*-GAL are described in [14]. Briefly, the UAS-*reaper* (*rpr*) transgene encodes the proapoptotic gene *reaper* and its expression under the control of GAL4 leads to cell death. The *dilp2*-GAL line is designated d2GAL and is specifically expressed in the mNSCs. Thus, d2GAL driven expression of UAS-*rpr* leads to ablation of the mNSCs. Two independent lines of UAS-*dilp2*RNAi (described below) were back crossed at least 6 times into *w^Dah^*. UAS-GFP-RNAi was obtained from Bloomington stock centre. FOXO^21^, FOXO^25^ and yw are described in [29]. UAS-InR^DN^ is described in [26] and was backcrossed into the *w^Dah^* background. Stocks were maintained and experiments conducted at 25°C on a 12h:12h light:dark cycle at constant humidity using standard sugar/yeast medium [24].

### Generation of UAS-dilp2RNAi constructs

Reactions were performed using Expanded High Fidelity Taq polymerase (Roche) on cDNA. A 400 bp fragment encompassing the whole of the *dilp2* sequence was obtained by PCR reaction whose DNA template was EST GH11579 (Fly Base). The PCR primers were: dilp2 forward (5′-ATGAGCAAGCCTTTGTCCTTC-3′), dilp2 reverse (5′-GACCACGGAGCAGTACTCCC-3′). To construct the inverted repeat, the “forward” fragment was firstly subcloned into BlueScriptII (Stratagene). The second fragment was inserted into the multi cloning site in the reverse orientation directly beside the “forward” fragment. As such direct reverse repeat sequences frequently tend to recombine within the two fragments in E. coli, we used SURE2 competent cells (Stratagene) to prevent recombination. The direct inverted repeat transgene was finally subcloned into the fly transformation vector pUAST. Purified UAS-dilp2RNAi construct was co-injected into *yw* eggs with pΔ2-3 helper plasmid using standard procedures. A single transformant line driven by d2GAL resulted in only a 60% reduction in *dilp2* transcript (data not shown). The construct was mobilised by crossing to a transposase expressing Δ2-3Sb line. The *dilp* levels in adult heads of nine independent insertion lines driven by d2GAL were analysed by RT-QPCR. Two lines chosen (UAS-dilp2RNAi A&B) gave 80% reduction in *dilp2*.

### Western Blots

Heads obtained from 40 females were homogenised in 20 µl of 50 mM Tris-HCl pH 7.5, 150 mM NaCl, 0.5 mM EGTA, 10% glycerol, 1% SDS and protease inhibitor cocktail (Sigma). Extracts were cleared by centrifugation and protein content determined with Bradford assay. 20 µg of total protein was separated on either 16% polyacrilamide Tris-Tricine-SDS gels as described in [30] for DILP2, or 12% Tris-Glycine-SDS gels for tubulin. The proteins were transferred to nitrocellulose membranes and probed for DILP2 (1∶5000, 15) or tubulin (1∶3500, monoclonal mouse clone DM1A, Sigma). The blots were revealed with HRP-conjugated secondary antibody and ECL reagent (Amersham).

### Quantitative RT-PCR

*dilp* transcript levels were measured as in [14], except SYBR Green master mix (Applied Biosystems) was used and five independent head RNA extractions performed per genotype.

### Lifespan

Procedures for lifespan studies were as described in [8] and [31]. Lifespan was measured in flies kept at 10/vial on standard food medium [24] and transferred to new food twice weekly. Deaths were scored 5 to 6 times in every 7 days.

### Fecundity

Fecundity of females in lifespan experiments was measured as described in [14]. Data are reported as the mean number of eggs laid per day per female±SEM over each 2 or 3 day period.

### Stress Tests

Mated females were generated and maintained as for lifespans. Survival of 100 females per genotype was measured on: (a) 5% hydrogen peroxide in 1.5% agar, 5% sucrose (oxidative stress), (b) 1% agar (starvation).

### Trehalose and Glucose Measurement

Flies were generated and maintained on 100 g/l sugar in standard lab food. Hemolymph was collected and pooled from either 12 adult flies after a 5 hour starvation by decapitation and centrifugation or from 5 late third instar wandering larvae. Glucose and trehalose in 1 µl of pooled liquid was measured as described in [14]. Whole fly trehalose was measured as described in [32] using the glucose assay above. Data are reported as mean±SEM.

### Lipid and Glycogen measurements

The glycogen and lipid contents of two adult female flies were measured 7 days post-eclosion as described in [33], [14]. Data are expressed relative to fresh body weight and reported as mean±SEM.

### Statistical analyses

Statistical analyses were performed using JMP (version 7) software (SAS Institute). Lifespan and stress data were subjected to survival analysis (Log Rank tests) and presented as survival curves. Other data were tested for normality using the Shapiro-Wilk W test on studentised residuals [34] and where appropriate log-transformed. One-way analyses of variance (ANOVA) were performed and planned comparisons of means were made using Tukey-Kramer HSD test. Data are presented as means of raw values± SEM, * denotes significant difference from controls (P<0.05).

### Identifying putative FOXO binding sites in *dilp2*, *3* and *5* promoters

The analysis was performed using Regulatory Sequence Analysis Tools [35] and perfect matches to the mouse Foxo1/Foxo4 binding sites [RWWAACA; 36] identified within 1 kb upstream of the ATG codon in dilp2, 3 or 5.

## Supporting Information

Figure S1 The effect of expression of a UAS-GFP RNAi transgene driven by d2GAL in the mNSCs on whole body trehalose content. Whole-fly trehalose content per mg of fly (fresh weight). N = 20 for all genotypes. There were no significant differences between genotypes showing that the trehalose phenotype of the dilp2RNAi flies was not due to non-specific effects of the RNAi machinery in the mNSCs.
(0.46 MB TIF)
