# Supplementary figures and images for: Reduction of DILP2 in Drosophila Triages a Metabolic Phenotype from Lifespan Revealing Redundancy and Compensation among DILPs
Source: PLoS One. 2008 Nov 13;3(11):e3721. doi: 10.1371/journal.pone.0003721 (PMC2579582; doi:10.1371/journal.pone.0003721)

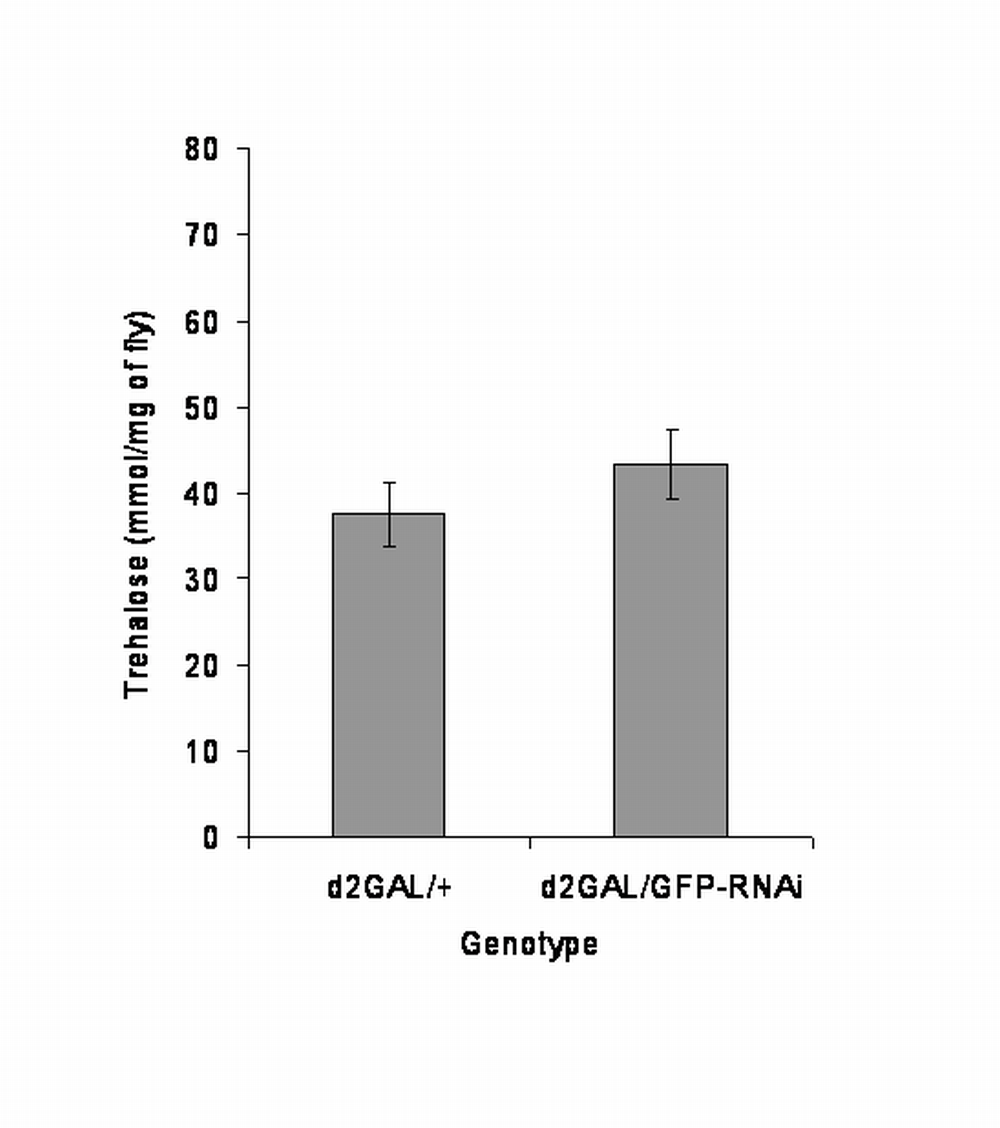

Supplement: Figure S1 — The effect of expression of a UAS-GFP RNAi transgene driven by d2GAL in the mNSCs on whole body trehalose content. Whole-fly trehalose content per mg of fly (fresh weight). N = 20 for all genotypes. There were no significant differences between genotypes showing that the trehalose phenotype of the dilp2RNAi flies was not due to non-specific effects of the RNAi machinery in the mNSCs. (0.46 MB TIF) [file pone.0003721.s001.tif]
